# Multiple Magnet Ingestion in a Child With an Overlooked Diagnosis Leading to Intestinal Perforation

**DOI:** 10.7759/cureus.104994

**Published:** 2026-03-10

**Authors:** Mohamed A Areed, Mohamed Elawdy, Ali Albalushi, Shahad s Almamari

**Affiliations:** 1 Pediatric Emergency Medicine, Sohar Hospital, Sohar, OMN; 2 Surgery, Sohar Hospital, Sohar, OMN

**Keywords:** delayed diagnosis, foreign body ingestion, intestinal perforation, multiple magnet ingestion, pediatrics

## Abstract

Foreign body (FB) ingestion is common in children below six years of age. Most FBs are passed spontaneously through the gastrointestinal tract. However, multiple magnetic ingestion increases the risk of intestinal obstruction and perforation via magnetic attraction through bowel walls. We report a case of a four-year-old boy who came with a delayed presentation of multiple magnet ingestion over a year before presentation to the emergency department. The diagnosis was overlooked in the first presentation by the primary health care doctor, and the FBs in the X-ray were interpreted as being an artifact. This case underscores the need for increased clinician awareness of having a high index of suspicion of FB ingestion in pediatric cases presenting with prolonged vague abdominal pain and to be vigilant upon reviewing radiological images.

## Introduction

Foreign body (FB) ingestion is a frequent pediatric emergency, particularly among children aged six months to six years [1]. FB ingestion is common in toddlers in Oman [2]. Most cases occur accidentally, with commonly ingested items including coins, toy components, sharp objects, batteries, and magnets [3]. Approximately 80-90% of gastrointestinal FBs pass spontaneously through the stomach, while 10-20% require endoscopic removal and fewer than 1% require surgical management to retrieve the object or address complications [4]. Among ingested objects, multiple magnets pose a distinct and serious risk. When more than one magnet is swallowed, magnetic attraction across adjacent bowel loops can occur, resulting in sustained compression of the intestinal walls. This may lead to pressure necrosis, obstruction, fistula formation, or perforation. Clinical presentation is often nonspecific, which can delay diagnosis and increase morbidity [5]. In our case, a four-year-old boy presented with prolonged nonspecific gastrointestinal symptoms. Diagnosis of multiple magnet ingestion was initially missed, leading to delayed management. We aimed to raise awareness of early suspicion of FB ingestion in children.

## Case presentation

A four-year-old boy presented with recurrent abdominal pain for one year. It was not associated with nausea or vomiting. The child had previously been evaluated by the primary physician one year prior to the same issue. An abdominal radiograph was assessed by the primary physician as showing an external artifact (zipper), and lactulose syrup was recommended for the treatment of constipation.

**Figure 1:**
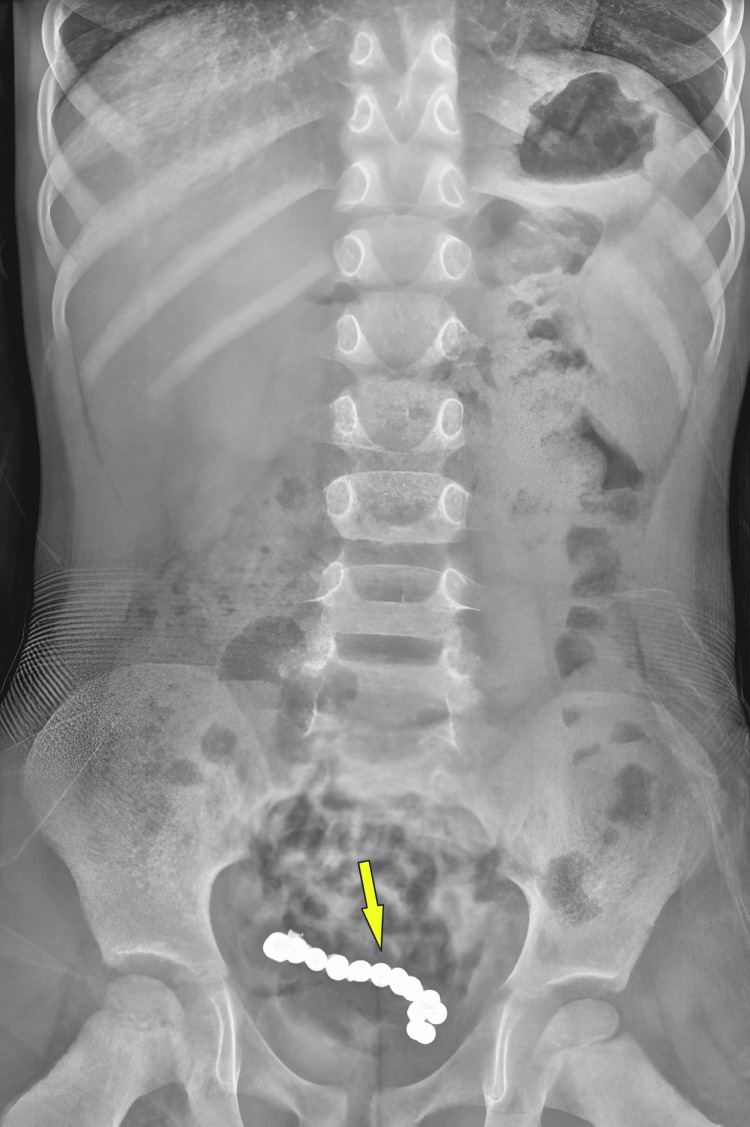
Abdominal radiograph in the erect position showing a well-defined chain of beaded magnets representing a foreign body (arrow)

**Figure 2:**
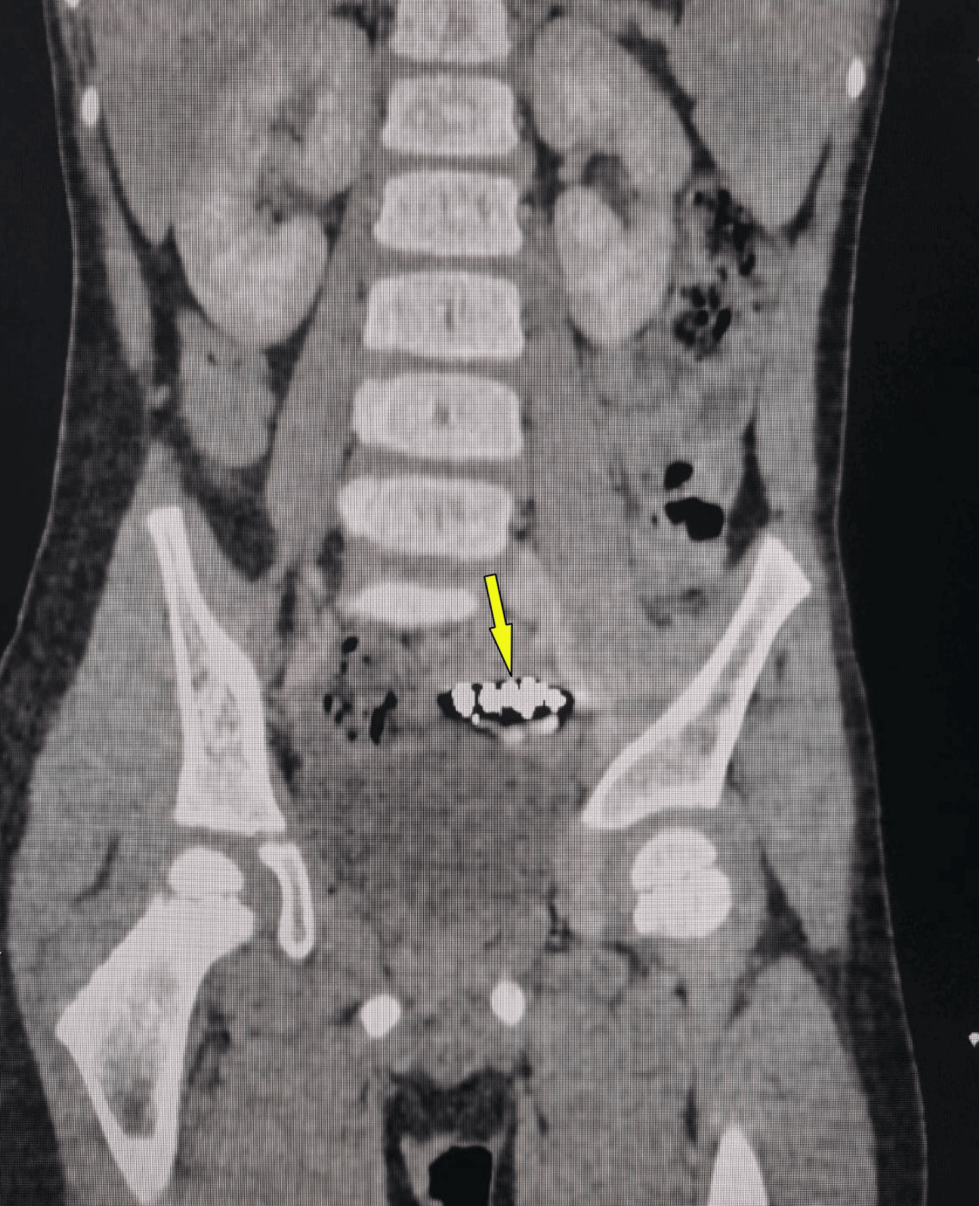
CT abdomen demonstrated a large, irregular FB (arrow) within the distal ileum FB: foreign body

Clinical exam revealed a soft abdomen without tenderness or guarding. Abdominal radiograph revealed a chain of radiopaque and rounded FBs in the lower abdomen (Figure 1). CT abdomen demonstrated a large, irregular FB within the distal ileum (Figure 2). Initial blood investigations were within normal range. Laparoscopy was planned for both diagnostic evaluation and definitive management.

**Figure 3:**
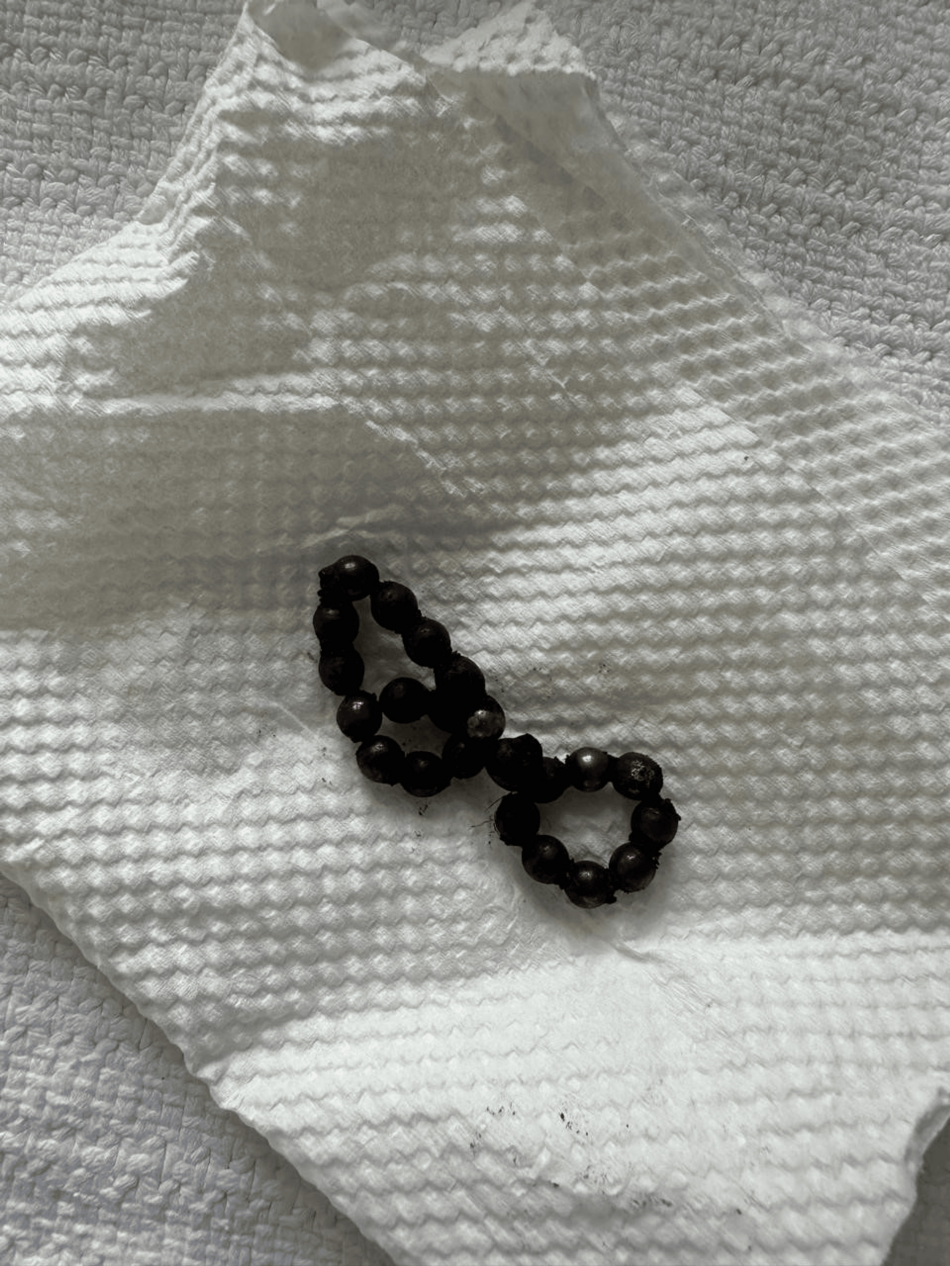
Foreign bodies after they were extracted

Surgical findings showed 22 pieces of magnet (Figure 3); the magnet was making a closed loop with adhesion. The bowel loop was necrotic because of FB, so the decision was to excise the affected segment. A 15 cm segment of mid-jejunum was resected, and an end-to-end anastomosis was performed. Histopathology confirmed the presence of a small bowel perforation, characterized by transmural defects with adjacent necrosis of the muscularis propria and focal granulation tissue formation. The child was discharged home on the fourth postoperative day in stable condition and was seen three months postoperatively with no complaint.

## Discussion

Infants and young children frequently explore their environment by placing objects in their mouths, which increases the risk of FB ingestion. Although FB ingestion is common in children, diagnosis may be challenging, particularly in unwitnessed events. As in our case, nonspecific clinical manifestations often contribute to delays in identification and treatment [6]. 

Approximately 80% of ingested FBs pass through the gastrointestinal tract without complications [7]. However, ingestion of multiple magnets poses the unique danger of being able to attract each other through different loops of bowel, arresting their movement, and potentially causing mural pressure necrosis. This can lead to bowel perforation, fistula formation, volvulus, obstruction, intra-abdominal sepsis, and death [8]. Among magnetic foreign objects, potent rare-earth neodymium magnets (commonly known as Buckyballs) pose a greater risk when compared to conventional magnets. In this instance, we dealt with traditional magnets. Nevertheless, 5 mm neodymium magnets can generate a force of up to half a kilogram when positioned in proximity to one another. The ingestion of such powerful multiple magnets can facilitate their alignment within the gastrointestinal tract within a timeframe of 12 to 48 hours, potentially leading to ischemia, necrosis, and perforation of the intervening intestinal walls [9].

Previous reports described similar presentations in young children. Jain et al. reported a two-year-old boy who presented two days after ingesting multiple magnets [10]. Mirza et al. described a comparable case involving a two-year-old girl who developed intestinal obstruction and pressure necrosis following multiple magnet ingestion [11]. Alareefy et al. reported a similar case of a four-year-old boy who ingested multiple magnets, who presented four months later. Thirteen magnetic pieces were removed with no obvious intestinal complications [12]. In most previously published cases, the interval between ingestion and presentation ranged from several days to weeks, which was shorter than the delayed presentation observed in our case.

Management of magnet ingestion depends on the number of magnets involved, their location within the gastrointestinal tract, and the presence of symptoms. In asymptomatic children, radiographic evaluation is necessary to determine whether the ingested object represents a single magnet, multiple magnets, or a magnet associated with another metallic FB [13]. In our case, the magnet seen on abdominal radiography was initially interpreted as an imaging artifact, which contributed to delayed management. This highlights the importance of maintaining a high index of suspicion when evaluating children with nonspecific gastrointestinal complaints.

Multiple magnets may adhere to one another and appear as a single object on imaging studies. Misinterpretation of multiple magnet ingestion as a solitary FB can delay treatment and increase the risk of complications. Computed tomography may be useful when perforation or other surgical complications are suspected [14].

Asymptomatic patients can be monitored with serial radiographs to assess the progression of the FB through the gastrointestinal tract. However, symptomatic children with one or more magnets in the digestive system, or those with a magnet ingested together with another metallic object, should be referred promptly for pediatric surgical evaluation and possible intervention [14].

According to the management approach proposed by Hussain et al., treatment of magnet ingestion depends on symptom status and magnet location. Early operative management is recommended when multiple magnets progress beyond the stomach, especially in symptomatic children, whereas endoscopic removal may be appropriate in selected asymptomatic cases. Endoscopic retrieval can be considered in carefully chosen asymptomatic patients when the magnets remain accessible within the upper gastrointestinal tract. This applies when imaging confirms that the magnets are still proximal to the pylorus and have not migrated distally. At this stage, significant mucosal injury is unlikely to have developed. Intervention should be undertaken promptly, ideally within the first 12-24 hours, to minimize the risk of progression and complications [15].

## Conclusions

Multiple magnet ingestions in children can manifest late and with vague gastrointestinal complaints. A high level of clinical suspicion and thorough radiologic assessment are therefore crucial for prompt diagnosis. The risk is greatest in boys, children less than six years, children with neurodevelopmental or behavioral conditions, including pica, and in older children with psychiatric disorders. Additional warning features include an unwitnessed choking event, sudden onset of coughing, gagging, drooling, or refusal to feed in a child who was previously well.
